# How leader-enforced positivity and humility–aspiration signaling shape emotional exhaustion and unethical behavior: evidence for distinct psychological pathways

**DOI:** 10.3389/fpsyg.2026.1774884

**Published:** 2026-05-13

**Authors:** Hussain Ali, Rong Zheng, Lingyun Meng, Muhammad Qasim

**Affiliations:** 1 School of International Trade and Economics, University of International Business and Economics, Beijing, China; 2 School of Economics and Management, Southwest Jiaotong University, Chengdu, Sichuan, China

**Keywords:** emotional exhaustion, humility–aspiration signaling, leader-enforced positive display rules, meaning-making, state suppression, unethical pro-self-behavior

## Abstract

This research examines how two contrasting leader signals—leader-enforced positive display rules (EPDR) and humility–aspiration signaling (HAS)—impact employees’ emotional exhaustion and unethical pro-self behavior. Drawing on social information processing theory, we propose a dual-pathway model in which EPDR leads to state suppression, whereas HAS fosters state meaning-making, which predicts emotional exhaustion and unethical pro-self behavior. Study 1 employed a time-lagged survey design with 489 full-time employees. Study 2 used a between-subjects vignette experiment with 276 full-time employees and experimentally manipulated EPDR versus HAS. The results showed that EPDR was positively related to state suppression, which predicted higher emotional exhaustion and unethical pro-self behavior. Conversely, HAS was positively related to state meaning-making, which predicted lower emotional exhaustion and unethical pro-self behavior. Dispositional optimism weakened the positive association between EPDR and state suppression, whereas hypercompetitive orientation weakened the positive association between HAS and state meaning-making. These findings demonstrate that EPDR can inadvertently deplete employees and encourage unethical pro-self behavior, whereas HAS can cultivate meaning-making and protect against these harmful outcomes, contingent on employees’ traits. Theoretical and practical implications are discussed.

## Introduction

Leaders are increasingly expected to cultivate positivity, sustain employee wellbeing, and maintain ethical conduct, particularly in contexts of uncertainty and continuous change. In this context, leadership styles inculcating positivity are explored and researched as a means of increasing social benefits (Redín et al., 2023) and enhancing ethical conduct and trust (Azila-Gbettor et al., 2024). However, some leaders not only express their own optimism but also require employees to “stay positive,” suppress negative reactions, and pretend not to be upset (Kaunang et al., 2025). We refer to such requirements as leader-enforced positive display rules (EPDR), which capture enforced positivity directed at employees. What remains unclear is whether these costs stem from positivity being enforced or from the normative signal that enforcement sends employees about what they are permitted to feel and express on the job (Diefendorff and Richard, 2003; Diefendorff et al., 2011). Addressing this question requires a contrasting signal, one whose normative content enables, rather than constrains, employees’ expressive and cognitive latitude.

EPDR refers to imposed emotional norms that require employees to maintain optimism, suppress negative emotions, and adhere to positive display expectations even when problems remain unresolved (Humphrey et al., 2015; Grandey and Melloy, 2017). As a social signal, EPDR communicates that emotional restraint and surface positivity are expected and rewarded, regardless of situational demands. While some studies evaluate how inappropriate emotional displays generate disadvantageous outcomes for the organization (Cheshin, 2020), other studies have investigated EPDR, indicating that when leaders enforce perpetual positivity and discourage employees from expressing their concern, such practices can jeopardize employee wellbeing, hinder ethical decision-making, and ultimately detrimentally affect organizational functioning and effectiveness (Chen G. et al., 2019; Qiu et al., 2024). Emotional suppression is linked to emotional exhaustion, depersonalization, and reduced personal accomplishment (Yin et al., 2023; Chang, 2020).

At the same time, research on leadership has placed increasing emphasis on humility as a strong alternative signal. Leadership behavior influences an individual’s or team’s perception of meaning, competence, and self-determination (Ma et al., 2023). Humility in leadership refers to leaders acknowledging limitations, seeking feedback, and recognizing others’ expertise, behaviors that signal openness to learning and respect for employees’ input (Zhong et al., 2020). Paradoxical leadership looks at the leadership humility phenomenon in parallel with leadership firmness to goals, as it suggests that leaders should have a warm and gentle approach toward employees, but they also need to be strong and relentless (Zhang et al., 2015). Relatedly, leader aspirational rhetoric, which involves articulating meaningful goals and instilling future-oriented purpose in employees, has been demonstrated to boost employee motivation and sense-making at work (Carton et al., 2014). However, these streams of leader humility and leader aspirational rhetoric have largely evolved in parallel in leadership studies, and research examining how humility and aspirational rhetoric may operate as a combined leadership signal remains limited.

To address this gap, we introduce humility–aspiration signaling (HAS) as a sequenced episode-level cue configuration within a single leader–employee interaction. In such episodes, a leader first acknowledges limitations and invites candid critique and then articulates a higher aspirational direction. HAS is not a leadership style. Humble leadership generally refers to an enduring pattern of expressed humility, including accurate self-view, appreciation of others, and teachability (Kelemen et al., 2023), whereas aspirational leadership emphasizes leader rhetoric that links daily work to higher-order goals and purpose (Carton et al., 2014). Paradoxical leadership reflects a broader orientation toward integrating competing yet interrelated behavioral demands (Zhang et al., 2015). HAS differs from adjacent leadership constructs in three important respects. First, it differs in its unit of analysis: humble leadership, aspirational leadership, and paradoxical leadership are typically theorized as more enduring behavioral tendencies or styles, whereas HAS concerns a bounded interaction. Second, it differs in its temporal logic: HAS is defined by the order in which the cues are delivered. Humility first broadens expressive latitude by signaling that candor is safe; aspiration then channels attention toward a higher-purpose direction. Third, it differs in its explanatory mechanism: HAS is not simply humility plus ambition, nor a generic blending of opposites. Its theoretical value lies in how the first cue changes the meaning of the second. Without humility, aspiration may be received as pressure; without aspiration, humility may create openness without direction. HAS, therefore, captures a specific signaling configuration not fully represented by existing constructs. Thus, our claim is not that HAS replaces existing leadership constructs but that it specifies a more proximal interactional mechanism through which leaders shape what employees infer and regulate in the moment.

The psychological mechanism linking the two components of HAS is sequential and interpretive rather than merely additive. When a leader first acknowledges limitations and invites candid critique, employees are likely to feel less interpersonally vulnerable and less compelled to protect themselves through caution, silence, or impression management. Research on leader humility suggests that such behavior reduces follower vulnerability, and work on psychological safety shows that lower interpersonal risk encourages more open engagement and learning-oriented responses (Oc et al., 2020). In such moments, employees are less likely to interpret the leader’s subsequent aspirational message as pressure to simply “stay positive” or display confidence. Instead, once defensiveness is reduced, the leader’s aspirational direction is more likely to be received as purposeful guidance that connects immediate demands to a broader goal. Because aspirational rhetoric helps frame ultimate goals and the larger meaning of present work, the sequence of humility followed by aspiration supports meaning-making rather than defensive compliance (Carton et al., 2014; Owens and Hekman, 2016).

This study draws on social information processing theory, which posits that individuals rely on social cues, particularly leader behaviors, to interpret situations, regulate emotions, and guide behaviors (Diefendorff and Richard, 2003; Salancik and Pfeffer, 1978). From a social information processing theory perspective, leadership behaviors function as salient social signals that define what is normative, acceptable, and valued in the workplace (Lu et al., 2019). Employees do not respond directly to objective events, but rather they interpret leader cues and adjust their psychological states accordingly.

In our present context, EPDR and HAS are two different social signals that influence employees’ psychological processing in opposite ways. EPDR signals that emotional restraint and surface positivity are normative and valued, even in the presence of unresolved problems (Chi and Grandey, 2019). Such cues shape employees’ interpretation of the situation as one in which emotional expression may violate implicit display norms. As a result, employees are likely to regulate emotions strategically by inhibiting authentic reactions. Within the logic of social information processing theory, this regulation manifests specifically as state suppression. Suppression directly reflects conformity to perceived emotional display expectations rather than broader threat-related stress responses (Ma et al., 2023). However, HAS communicates humility and aspirational goals. These behaviors signal that reflection, openness, and goal striving are appropriate responses to workplace challenges (Carton et al., 2014; Naseer et al., 2020; Fan et al., 2022). HAS provides interpretive cues that link current work demands to higher-purpose goals. Hence, HAS promotes state meaning-making, a cognitive process occurring in a particular context that aids individuals in comprehending their work experiences by providing a distinct sense of purpose and value (Allan et al., 2019; Rosso et al., 2010).

In organizational practice, leaders facing setbacks, deadlines, or uncertainty often attempt to sustain forward movement in one of two ways. One approach is to press employees to remain upbeat and adhere to positive display expectations despite unresolved problems (Diefendorff and Richard, 2003). Another is to acknowledge difficulty candidly while directing employees toward a larger aspirational direction (Carton et al., 2014; Oc et al., 2020; Edmondson, 1999). These alternatives are not merely stylistic differences; they communicate distinct normative messages about what is appropriate for employees to feel, express, and do in the moment (Diefendorff and Richard, 2003; Salancik and Pfeffer, 1978). Examining EPDR and HAS together, therefore, enables us to isolate how leaders’ signals with opposing normative content shape employees’ immediate regulatory responses. The comparison matters not because both are ostensibly “positive” leadership responses but because both are plausible responses to organizational strain that differ in the latitude they afford for authenticity and interpretation.

Accordingly, we propose a dual-pathway model in which leader signals channel employees into distinct modes of internal regulation (state suppression and state meaning-making) with subsequent consequences for wellbeing and ethical conduct (emotional exhaustion and unethical pro-self behavior). Emotional exhaustion constitutes the primary aspect of burnout, occurring when individuals experience a depletion of energy and an overwhelming emotional strain (Chen K. -Y. et al., 2019; Costin et al., 2023; Wu et al., 2020). Unethical pro-self behavior occurs when individuals act contrary to their moral principles for personal gain, such as deceit or rule manipulation (Vriend et al., 2020; Bryant and Merritt, 2021).

Social information processing theory asserts that social signals are not universally interpreted; personal attributes affect how employees perceive and process signals from leaders (Lu et al., 2019). We focus on two traits that are conceptually aligned with our dual paths. Dispositional optimism, defined as a general expectancy of positive outcomes, influences individuals’ perceptions of adverse or authoritative signals (Li et al., 2019). Hypercompetitive orientation, characterized by an intense need to surpass others and achieve victory at any cost, influences individuals’ reactions to aspirational stimuli (Yoon et al., 2020). These traits matter not because they affect outcomes but because they shape how leaders’ signals are interpreted. Hence, we analyze how these traits play the moderating role in our dual pathways.

This study contributes in several ways. First, it shifts the focus from leadership styles to leader signals at the episode level. It shows that the informational content of a signal (rather than its emotional tone) determines which regulatory pathway employees take. Studying EPDR and HAS together is crucial, as it allows us to pinpoint signal informativeness as the mechanism underlying state suppression versus state meaning-making (Diefendorff and Richard, 2003; Salancik and Pfeffer, 1978; Morgeson et al., 2015). Second, this research connects state suppression and state meaning-making to emotional exhaustion and unethical pro-self behavior, thereby integrating ethics and wellbeing into a single framework. Third, it highlights how dispositional optimism and hypercompetitive orientation influence these effects at the individual level. These findings explain why EPDR and HAS do not affect all employees in the same way.

### Theoretical background and hypothesis

Social information processing theory explains how leader signals shape employee responses. This theory posits that individuals interpret social cues (leader behaviors) to construct norms and to make sense of situations, events, and their own behavior (i.e., what is appropriate, valued, and expected) (Diefendorff and Richard, 2003; Salancik and Pfeffer, 1978; Wang et al., 2022; Boekhorst, 2015). Leaders set the standards for behavior, values, and expectations in the workplace (Cao et al., 2020). Leaders shape employees’ views on norms related to emotions, expression, and performance through regular interactions and discussions. These norms subsequently affect attitudes and conduct (Wang et al., 2022; Zeng et al., 2020). EPDR and HAS are considered distinct social signals that convey different information about emotional expression and the importance of work. Employees respond through state suppression when EPDR emphasizes emotional restraint and positive display expectations, signaling that authentic expression may be inappropriate. Employees also engage in state meaning-making when HAS signals humility and aspirational directions, leading them to interpret work demands as purposeful and value-laden. These proximal responses shape emotional exhaustion and unethical pro-self behavior.

In our model, we specify the primary cue–mediator matching, rather than the absolute exclusivity of cross-path associations. As EPDR communicates restrictive display norms, we theorize state suppression to be its most proximal regulatory response. Moreover, as HAS communicates openness and higher-purpose direction, we theorize state meaning-making to be its most proximal interpretive response. However, HAS may also reduce suppression because cues of humility and candid invitation make defensive restraint less necessary.

### EPDR and state suppression

EPDR refers to norms communicated to employees, which include maintaining a cheerful demeanor, concealing negative emotions, and refraining from expressing dissatisfaction (Humphrey et al., 2015). Research on emotional labor suggests that display rules can suppress authentic emotional expression and hinder open discussions about concerns or problems (Hong and Kim, 2023; Lee, 2018). In the context of our study, when leaders consistently assert that negative emotions or dissent are unacceptable, employees feel compelled to suppress their negative reactions (the primary targets of EPDR display norms) to maintain a favorable standing within the team. Display rules are associated with increased emotional regulation effort (Humphrey, 2023). Based on these arguments and in line with social information processing theory, we propose the following hypothesis:

*H1: EPDR is positively related to state suppression.*

### HAS and state meaning-making

HAS refers to a leadership signal in which leaders openly acknowledge their limitations while also establishing meaningful and aspirational goals for the group to pursue. Recent research suggests that leaders who display humility foster openness, learning, and enhanced interpersonal interactions by acknowledging their fallibility and showing consideration for others (Kelemen et al., 2023; Hadmar et al., 2022). Similarly, research on meaningful work indicates that when employees view their responsibilities as aligned with broader principles and contributions, they experience increased purpose and engagement (Allan et al., 2019; Schnell and Hoffmann, 2020; Lozovetska et al., 2024). Integrating humility with a goal-oriented approach is likely to encourage employees to consider the significance of their roles rather than experiencing threat.

HAS activates state meaning-making through a sequence of cues. The humility component reduces follower vulnerability and establishes psychological safety for open engagement (Edmondson, 1999). With such safety established, the aspiration component functions as an interpretive cue that connects current work demands to higher-purpose goals (Carton et al., 2014). This cognitive connection to a transcendent purpose constitutes meaning-making (Allan et al., 2019; Rosso et al., 2010).

We assert that these episodes promote state meaning-making through a sequenced cognitive process. The humility component reduces follower vulnerability by signaling that candid expression is welcomed and fallibility is acknowledged. This allows the aspiration component to be received as purposive direction that links current demands to higher-purpose goals (Carton et al., 2014), thereby facilitating state meaning-making rather than simply motivation or engagement. This represents the most proximal regulatory response to HAS (Allan et al., 2019; Rosso et al., 2010). Accordingly, we hypothesize that:

*H2: Humility–aspiration signaling is positively related to state meaning-making.*

### State suppression, emotional exhaustion, and unethical pro-self behavior

We select state suppression as the mediator for Pathway 1 because it is the most proximal regulatory response to constraining display norms—directly reflecting conformity to display expectations rather than broader threat or stress responses (Ma et al., 2023). We select state meaning-making as the mediator for Pathway 2 because it captures the specific cognitive work of constructing purpose from enabling interpretive cues—rather than general motivation or engagement (Allan et al., 2019; Rosso et al., 2010).

State suppression creates significant psychological burdens due to the necessity for constant self-regulation and the restriction of opportunities for emotional recovery (Yun et al., 2019). Recent research indicates that heightened emotional labor demands and the suppression of emotional states are associated with increased emotional exhaustion and deteriorated mental health among employees (Humphrey, 2023; Chen et al., 2022; Kern et al., 2021). Exhaustion represents a condition of diminished energy and emotional distress, which reflects the core component of burnout. State suppression has moral consequences because it affects individuals’ wellbeing. Individuals are more likely to engage in unethical behavior that serves their self-interest when their self-regulatory capacity is weakened (Storme et al., 2021; Wang, 2022).

We assume that under these conditions, employees may prioritize personal benefit and convenience over internalized ethical ideals, thereby increasing the likelihood of unethical pro-self behavior. Accordingly, we propose that state suppression is an unfavorable psychological mechanism linking EPDR to emotional exhaustion and unethical conduct, and we advance the following hypothesis:

*H3a: State suppression is positively related to emotional exhaustion.*

*H3b: State suppression is positively related to employees’ unethical pro-self behavior.*

### State meaning-making, emotional exhaustion, and unethical pro-self behavior

State meaning-making is a state in which employees perceive their work and efforts as meaningful and valuable. Extant research on meaningful work indicates that a heightened state of meaning-making is associated with increased motivation and decreased emotional exhaustion, as meaningfulness fosters resilience and helps employees manage their emotions (Allan et al., 2019; Lysova et al., 2019; Bailey et al., 2019). In the ethical context, when employees perceive their work as morally meaningful and valuable, they are more likely to act in ways that are consistent with their moral values and long-term goals. They are also less likely to engage in unethical pro-self behavior for short-term personal gain (Rai et al., 2023). Moreover, meaningful work is associated with positive emotional outcomes and greater satisfaction among helping professionals (Rai et al., 2023). We assume that state meaning-making is an adaptive psychological pathway through which HAS reduces emotional exhaustion and unethical pro-self behavior among employees. Accordingly, we propose the following hypotheses:

*H4a: State meaning-making is negatively related to emotional exhaustion.*

*H4b: State meaning-making is negatively related to employees’ unethical pro-self behavior.*

### Dispositional optimism as a moderator

Dispositional optimism refers to employees’ general expectation of positive outcomes, and it can shape how employees respond to social signals. Extant studies indicate that optimism correlates positively with both subjective and psychological wellbeing of employees over time, partly because optimistic individuals perceive challenges as less threatening and more manageable (Pérez et al., 2021). High dispositional optimism is also correlated with more adaptive emotional and behavioral tendencies in the workplace (Li et al., 2019; Scheier et al., 2021). Building on these findings, we believe that dispositionally optimistic employees will perceive the EPDR differently and not as highly constraining.

**Figure 1:**
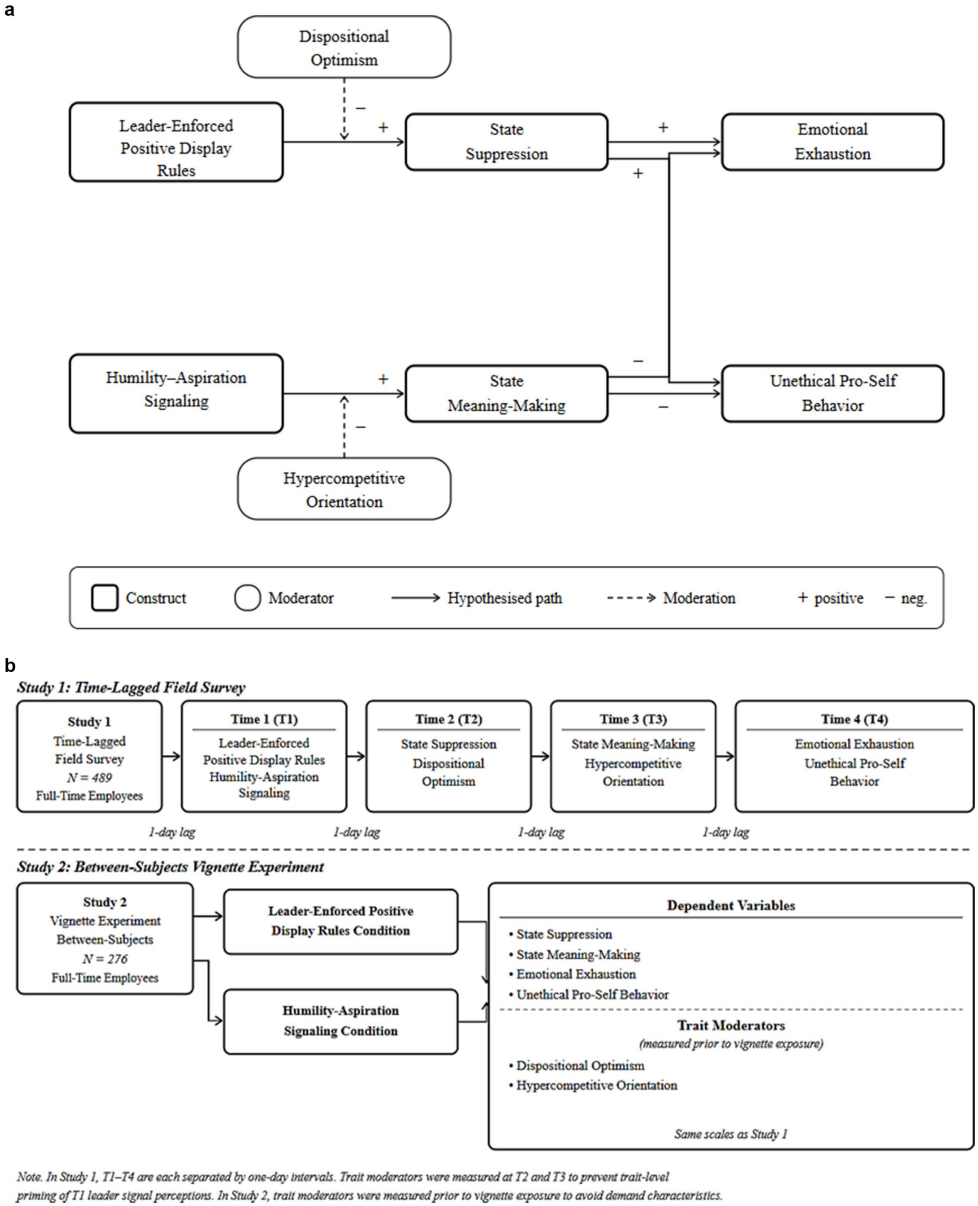
**(a)** Overall conceptual framework. **(b)** Overview of research design. The figure summarizes the sequence, sample sizes, manipulations, measurements, and moderation tests across Study 1 and Study 2.

We assign dispositional optimism to Pathway 1 because its primary mechanism, threat reappraisal (Nes and Segerstrom, 2006), is proximal to EPDR’s controlling content but not to HAS’s enabling cues. We assign a hypercompetitive orientation to Pathway 2 because its primary mechanism, competitive framing of social interactions (Orosz et al., 2018; Song, 2021), is proximal to HAS’s aspirational content but not to EPDR’s constraining content. Therefore, we propose the following hypotheses (see Figure 1a):

*H5a: Dispositional optimism weakens the relationship between EPDR and state suppression.*

*H5b: EPDR has positive indirect effects on (i) emotional exhaustion and (ii) employees’ unethical pro-self behavior via state suppression.*

### Hypercompetitive orientation as a moderator

Hypercompetitive orientation refers to employees’ tendency to view social interactions through a highly competitive lens and to seek to outperform others even at high personal and relational cost. Extant studies conceptualize hypercompetitive orientation as multidimensional and link it to more self-centered and conflict-prone behavior (Orosz et al., 2018). Other research indicates that hypercompetitive attitudes can exacerbate detrimental workplace outcomes, such as harassment, suggesting that intense competitive drives may intensify self-protective or aggressive behaviors (Song, 2021). Hypercompetitive orientation among employees is also associated with highly competitive environments characterized by elevated unethical behavior. Hence, in our research, we propose that hypercompetitive orientation moderates the relationship between HAS and state meaning-making, such that employees in highly competitive environments may be less responsive to HAS in terms of its effect on state meaning-making. Therefore, we propose the following:

*H6a: Hypercompetitive orientation weakens the relationship between HAS and state meaning-making.*

*H6b: HAS has negative indirect effects on (i) emotional exhaustion and (ii) employees’ unethical pro-self behavior via state meaning-making.*

## Methods

### Overview of the research design

To assess the hypothesized dual-pathway model and enhance confidence in the results, we employed a two-study design using divergent methodological techniques consistent with best research practice for theory testing in organizational research (Ployhart and Vandenberg, 2010). Figure 1b provides an overview of our research design to clarify the sequencing and comparative logic of our two studies. Study 1 utilized a multi-wave, time-lagged field survey of full-time employees to examine the relationships between EPDR and HAS, alongside the mediating effects of state suppression and state meaning-making, as well as the moderating influences of dispositional optimism and hypercompetitive orientation. Study 2 employed a between-subjects vignette experiment to determine the causal effects of the two leader signals and to replicate Study 1.

### Methods: Study 1

#### Sample and procedure

Study 1 employed a multi-wave, time-lagged field survey design. We recruited full-time employees from a professional online panel provider and explained the research purpose, survey procedures, and confidentiality assurances before participation. Eligible participants were required to be employed full time and working in a team-based setting. Data were collected across four waves to reduce common method bias and to map the causal sequence implied by social information processing theory (Diefendorff and Richard, 2003; Salancik and Pfeffer, 1978).

At Time 1 (T1), participants reported their perceptions of HAS, EPDR, and demographic information. At Time 2 (T2), conducted 1 day after T1, participants completed measures of state suppression and dispositional optimism. At Time 3 (T3), 1 day after T2, participants reported state meaning-making and hypercompetitive orientation. At Time 4 (T4), 1 day after T3, participants completed measures of emotional exhaustion and self-serving unethical intentions. One-day lags were used because episode-level regulatory responses emerge and dissipate within short temporal windows, emotional exhaustion can vary meaningfully at the daily level, and longer intervals would increase the likelihood that subsequent leader interactions contaminate the focal episode-to-response linkage (Humphrey, 2023; Poetz and Volmer, 2022; Basinska and Gruszczynska, 2020). We also used 1-day intervals because state suppression and state meaning-making were theorized as short-term responses to recent leader cues rather than enduring orientations. A short lag, therefore, preserves the episode-level nature of these processes while also introducing temporal separation between the predictor, mediator, and outcome measures. This approach is consistent with diary and daily organizational research, which is well suited to capturing short-term work processes and everyday work experiences, including day-level leadership dynamics (Ohly et al., 2010). Trait moderators (T2 and T3) were not positioned at T1 to prevent trait-level priming from inflating associations with simultaneously reported leader signal perceptions.

A total of 489 participants provided complete responses across all four waves, yielding the final matched sample. The average age of participants was 37.20 years (SD = 5.09), and 44.4% were female. With respect to organizational tenure, 26.6% had 1–3 years of tenure, 31.1% had 4–8 years, 22.1% had 9–14 years, 14.5% had 15–19 years, and 5.7% had more than 20 years. Regarding education, 34.8% held a bachelor’s degree, 43.4% held a master’s degree, and 21.9% held a doctoral degree.

Importantly, the present theory concerns employees’ inferences from bounded leader episodes rather than broad evaluations of enduring leadership style. Accordingly, we operationalized EPDR and HAS as interactional signals: in Study 1, all leader-perception items were prefaced with “Recently, my supervisor …” to anchor responses to recent behaviors and discrete episodes.

#### Measures

All focal constructs were measured using 7-point Likert scales (1 = *strongly disagree*, 7 = *strongly agree*). Leader-perception items referenced specific recent behaviors to anchor responses to discrete episodes rather than dispositional tendencies (Hansbrough et al., 2021). We use the prefix *state* to indicate that suppression and meaning-making are situationally elicited regulatory processes rather than stable dispositions. Moreover, to reinforce the episodic framing, all leader-perception items were prefaced with “Recently, my supervisor.”

*Leader-enforced positivity* was assessed using a 4-item scale adapted from Diefendorff et al. (2005). A sample item is, “Recently, my leader forces a positive atmosphere, even when problems arise.” (*α* = 0.86).

*Humility–aspiration signaling* was assessed using an 8-item scale adapted from Owens et al. (2013) and Zhang et al. (2015). A representative item is, “Recently, my leader shows humility and a drive to succeed” (*α* = 0.89).

*State suppression*: Participants completed the 4-item suppression subscale from the Emotion Regulation Questionnaire (Gross and John, 2003). A sample item is, “I control my emotions by not expressing them” (*α* = 0.84).^1^

*State meaning-making:* We used a 4-item scale adapted from Steger et al. (2006) to capture work-related meaning constructed in response to the leader’s behavior, with item wording contextualized to employees’ work experience. A sample item is, “I have a clearer sense of why this work is worth doing” (*α* = 0.90).

*Emotional exhaustion:* We assessed resource depletion using the 3-item short form of the Maslach Burnout Inventory (Maslach and Jackson, 1981). A sample item is, “I feel emotionally drained from my work” (*α* = 0.91).

*Unethical pro-self behavior*: Participants reported behavioral intentions using four items from Umphress et al. (2010). A sample item is, “I would lie to get a personal advantage at work” (*α* = 0.86).

*Dispositional optimism:* Trait optimism was measured using the 6-item Life Orientation Test—Revised (Scheier, 1994). A sample item is, “In uncertain times, I usually expect the best” (*α* = 0.82).

*Hypercompetitive orientation:* Participants completed the 3-item short form of the Hypercompetitive Attitude Scale (Ryckman et al., 1990). A sample item is, “Winning in competition makes me feel more powerful as a person” (*α* = 0.85).

### Methods: Study 2

#### Sample and procedure

Study 2 employed a between-subjects experimental design to complement the time-lagged survey approach used in Study 1 and to strengthen causal inference regarding leader signaling effects. We conducted an online vignette experiment in which participants were randomly assigned to one of two conditions: EPDR or HAS. An *a priori* power analysis using G*Power (Faul et al., 2009) indicated that a minimum sample size of 210 participants was required to detect a medium effect size (*f* = 0.25) with a significance level of *α* = 0.05 and statistical power of 0.95. To account for potential exclusions, we recruited 280 full-time employees through a professional online panel provider. After excluding four participants who failed both attention checks, the final sample consisted of 276 participants.

Participants were required to be employed full time, have at least 6 months of organizational tenure, and work in a team-based setting. The final sample was 52.5% female, with a mean age of 35.53 years (SD = 7.2). Regarding education, 35.1% of participants held a bachelor’s degree, 42.0% held a master’s degree, and 22.8% held a doctoral degree. With respect to organizational tenure, 12.3% of participants had 1–3 years of tenure, 39.1% had 4–8 years, 27.2% had 9–14 years, 16.3% had 15–19 years, and 5.1% had more than 20 years. Participants received monetary compensation consistent with the panel provider’s standard incentive practices. Participation was open across industries to enhance the generalizability of the findings.

In Study 2, the episodic nature of EPDR and HAS was reinforced through a single meeting vignette. Because participants responded to one bounded leader–employee interaction rather than an ongoing supervisory relationship, the focal explanatory variable was the content of the immediate leader signal rather than a generalized impression of leadership style.

#### Experimental design

Before the study commenced, participants reported demographic information and completed attention check questions during the survey. They were then randomly assigned to either the EPDR condition or the HAS condition. Participants first completed the dispositional optimism and hypercompetitive orientation measures, and then subsequently completed a vignette-based scenario experiment. In the scenarios, they were instructed to imagine themselves as an employee attending a team meeting at a critical stage of a project. In the HAS condition, the supervisor acknowledged limitations, invited critical feedback, and articulated a specific, aspirational next step, whereas in the EPDR condition, the supervisor emphasized maintaining a positive tone and discouraged discussion of problems (see Appendix A). Following the scenario, participants completed manipulation checks and measures of state suppression, state meaning-making, emotional exhaustion, and unethical pro-self-behavior (the same scales were used as in Study 1). All items were rated on a 7-point Likert scale (1 = strongly disagree to 7 = strongly agree).

#### Analytic strategy

We tested the hypotheses in three stages. First, we evaluated manipulation effectiveness and scenario realism via mean comparisons across conditions. Second, we tested the dual-pathway mediation model using PROCESS Model 4 with 10,000 bootstrap samples and bias-corrected confidence intervals. The condition was specified as the independent variable, with state suppression and state meaning-making modeled as parallel mediators predicting emotional exhaustion and unethical pro-self behavioral intentions in separate models. Third, we tested moderated mediation using PROCESS Model 7.

## Results and analysis

### Results and analysis: Study 1

A series of confirmatory factor analyses verified construct distinctiveness. The hypothesized eight-factor model (EPDR, HAS, state suppression, state meaning-making, emotional exhaustion, unethical pro-self behavior, dispositional optimism, hypercompetitive orientation) fit the data well, *χ*^2^ (566) = 1030.75, *χ*^2^/df = 1.82, CFI = 0.97, TLI = 0.96, RMSEA = 0.04, RMR = 0.04. All alternative models fit significantly worse. The seven-factor model combining EPDR and HAS, *χ*^2^ (573) = 2608.37, CFI = 0.85, and the five-factor model combining all leader and trait variables, *χ*^2^ (584) = 4757.36, CFI = 0.69, both showed poorer fit. The one-factor and common method models fit very poorly (CFIs < 0.40, RMSEA > 0.15) (see Table 1).

**Table 1:** Model fitness indices.

Model	*χ* ^2^	df	*χ*^2^/df	CFI	TLI	RMSEA	RMR
Baseline eight-factor model (EPDR, HAS, SS, SM, EE, UPB, DO, and HO)	1030.75	566	1.82	0.97	0.96	0.04	0.04
Seven-factor model (EPDR + HAS)	2608.37	573	4.55	0.85	0.83	0.08	0.08
Five-factor model (EPDR + HAS + DO + HO)	4757.36	584	8.15	0.69	0.67	0.12	0.13
Three-factor model (EPDR + HAS + DO + HO, SS + SM, EE, UPB)	5968.71	588	10.15	0.60	0.57	0.14	0.16
One-factor model	9668.35	601	16.08	0.33	0.19	0.17	0.21
Common method factor model	8,739	594	14.71	0.39	0.36	0.17	0.17

EPDR, leader-enforced positive display rules; HAS, humility–aspiration signaling: SS, state suppression; SM, state meaning-making; EE, emotional exhaustion; UPB, unethical pro-self behavior; DO, dispositional optimism; HO, hypercompetitive orientation.

### Correlation analysis

Table 2 presents the means, standard deviations, and correlations among study variables. Mean scores ranged from 3.63 to 4.81 (SD = 1.15–1.82), reflecting moderate endorsement levels on the seven-point scale. The correlations were consistent with theoretical expectations. Leader-enforced positivity correlated positively with state suppression (*r* = 0.32, *p* < 0.01), emotional exhaustion (*r* = 0.18, *p* < 0.01), and unethical pro-self behavior (*r* = 0.25, *p* < 0.01). HAS correlated positively with state meaning-making (*r* = 0.29, *p* < 0.01) and negatively with suppression (*r* = −0.31, *p* < 0.01), exhaustion (*r* = −0.11, *p* < 0.05), and unethical behavior (*r* = −0.29, *p* < 0.01). Suppression correlated positively with both exhaustion (*r* = 0.37, *p* < 0.01) and unethical behavior (*r* = 0.47, *p* < 0.01), whereas meaning-making correlated negatively with these outcomes (*r* = 0.23–0.27, *p* < 0.01). As expected, dispositional optimism related positively to HAS (*r* = 0.24, *p* < 0.01) and meaning-making (*r* = 0.17, *p* < 0.01) but negatively to suppression (*r* = −0.20, *p* < 0.01) and EPDR (*r* = −0.13, *p* < 0.01). Hypercompetitive orientation showed the opposite pattern, correlating negatively with HAS (*r* = −0.32, *p* < 0.01) and meaning-making (*r* = −0.29, *p* < 0.01) and positively with suppression (*r* = 0.27, *p* < 0.01), exhaustion (*r* = 0.22, *p* < 0.01), and unethical behavior (*r* = 0.12, *p* < 0.01).

**Table 2:** Means, standard deviations, and correlations.

Variables	Mean	SD	(1)	(2)	(3)	(4)	(5)	(6)	(7)	(8)
Leader-enforced positivity	3.98	1.49	1							
Humility–aspiration signaling	4.13	1.82	−0.05	1						
State suppression	4.40	1.33	0.32^**^	−0.31^**^	1					
State meaning-making	4.21	1.50	−0.030	0.29^**^	−0.27^**^	1				
Dispositional optimism	4.81	1.15	−0.13^**^	0.24^**^	−0.20^**^	0.17^**^	1			
Hypercompetitive orientation	3.63	1.60	0.072	−0.32^**^	0.27^**^	−0.29^**^	−0.20^**^	1		
Unethical pro-self behavior	4.61	1.29	0.25^**^	−0.29^**^	0.47^**^	−0.27^**^	−0.18^**^	0.12^**^	1	
Emotional exhaustion	4.21	1.34	0.18^**^	−0.11^*^	0.37^**^	−0.23^**^	−0.09^*^	0.22^**^	0.22^**^	1
Sex	1.44	0.49	0.00	−0.09^*^	0.07	0.03	−0.06	0.05	0.06	0.03
Age	37.20	5.09	0.09	0.03	0.01	−0.01	0.04	0.00	0.05	−0.05
Education	1.87	0.74	0.05	−0.05	0.06	−0.01	0.03	0.02	−0.03	0.01
Tenure	8.46	6.35	0.04	−0.05	0.09^*^	−0.06	−0.05	0.01	0.02	0.09

*N* = 489. Cronbach’s alphas are shown in parentheses on the diagonal.

Sex: 0 = male, 1 = female. Education: 1 = bachelor’s degree; 2 = master’s degree; 3 = doctoral degree.

^*^p < 0.05, ^**^p < 0.01.

### Regression analysis

We tested the proposed indirect effects of leader-EPDR on employees’ emotional exhaustion and unethical pro-self behavior through state suppression, as well as the moderating role of dispositional optimism on the EPDR–suppression link.

As shown in Table 3, EPDR significantly predicted state suppression (*β* = 0.32, SE = 0.04, *p* < 0.001), supporting H1. In turn, state suppression was positively associated with both unethical pro-self behavior (*β* = 0.38, SE = 0.04, *p* < 0.001) and emotional exhaustion (*β* = 0.30, SE = 0.05, *p* < 0.001), supporting H3a and H3b. The indirect effect of EPDR on unethical behavior via suppression was significant (*Effect* = 0.12, 95% CI [0.08, 0.17]), as was the indirect effect on emotional exhaustion (*Effect* = 0.09, 95% CI [0.05, 0.14]), supporting H5a. Importantly, the interaction between EPDR and dispositional optimism was significant (*β* = −0.09, SE = 0.03, *p* = 0.001), indicating that optimism weakened the positive relationship between EPDR and suppression, consistent with H5a.

**Table 3:** Leader-enforced positivity effects.

Variables	State meaning-making			State suppression			Unethical pro-self behavior			Emotional exhaustion		
	*β*	SE	*p*	*β*	SE	*p*	*β*	SE	*p*	*β*	SE	*p*
Sex	0.03	0.13	0.46	0.07	0.11	0.09	0.03	0.10	0.41	0.00	0.11	0.96
Age	−0.02	0.01	0.73	−0.01	0.01	0.74	0.04	0.01	0.33	−0.06	0.01	0.16
Education	−0.01	0.09	0.83	0.04	0.08	0.30	−0.06	0.07	0.11	−0.01	0.08	0.85
Tenure	−0.07	0.01	0.14	0.08	0.01	0.08	−0.03	0.01	0.50	0.05	0.01	0.25
Leader-enforced positivity	−0.03	0.05	0.54	0.32	0.04	0.00	0.12	0.04	0.00	0.08	0.04	0.07
State suppression							0.38	0.04	0.00	0.30	0.05	0.00
State meaning-making							−0.17	0.04	0.00	−0.15	0.04	0.00
Leader-enforced positivity × dispositional optimism				−0.09	0.03	0.00						

Path	Effect	BootSE	BootLLCI	BootULCI
Leader-enforced positivity → state suppression → unethical pro-self behavior	0.12	0.02	0.08	0.17
Leader-enforced positivity → state suppression → emotional exhaustion	0.09	0.02	0.05	0.14

*N* = 489. PROCESS macro by Hayes was used; Model 4 was used for mediation analyses, and interaction effects were estimated using Model 1. SE = Standard error; bootstrap samples = 10,000.

As shown in Table 4, HAS positively predicted state meaning-making (*β* = 0.30, SE = 0.04, *p* < 0.001), supporting H2, and negatively predicted state suppression (*β* = −0.30, SE = 0.03, *p* < 0.001). State meaning-making was negatively associated with both unethical pro-self behavior (*β* = −0.13, SE = 0.04, *p* < 0.01) and emotional exhaustion (*β* = −0.15, SE = 0.04, *p* < 0.001), supporting H4a and H4b. The indirect effects were significant: HAS indirectly reduced unethical behavior through meaning-making [*Effect* = −0.04, 95% CI (−0.07, −0.01)] and indirectly reduced emotional exhaustion through meaning-making [*Effect* = −0.04, 95% CI (−0.08, −0.02)], supporting H6a. The interaction between HAS and hypercompetitive orientation was significant (*β* = −0.07, SE = 0.01, *p* < 0.001), indicating that hypercompetitive orientation weakened the positive relationship between HAS and meaning-making, consistent with H6b.

**Table 4:** Effects of humility–aspiration signaling.

Variables	State suppression			State meaning-making			Unethical pro-self behavior			Emotional exhaustion		
	*β*	SE	*p*	*β*	SE	*p*	*β*	SE	*p*	*β*	SE	*p*
Sex	0.05	0.12	0.27	0.06	0.13	0.19	0.02	0.10	0.66	0.00	0.12	0.97
Age	0.02	0.01	0.60	−0.02	0.01	0.63	0.05	0.01	0.17	−0.05	0.01	0.20
Education	0.04	0.08	0.33	0.01	0.09	0.86	−0.07	0.07	0.09	0.00	0.08	0.91
Tenure	0.08	0.01	0.08	−0.05	0.01	0.24	−0.03	0.01	0.50	0.05	0.01	0.23
Humility–aspiration signaling	−0.30	0.03	0.00	0.30	0.04	0.00	−0.14	0.03	0.00	0.04	0.03	0.73
State suppression							0.39	0.04	0.00	0.33	0.05	0.00
State meaning-making							−0.13	0.04	0.00	−0.15	0.04	0.00
Humility–aspiration signaling × hypercompetitive orientation				−0.07	0.01	0.00						

Path	Effect	BootSE	BootLLCI	BootULCI
Humility–aspiration signaling → state meaning-making → unethical pro-self behavior	−0.04	0.01	−0.07	−0.01
Humility–aspiration signaling → state meaning-making → emotional exhaustion	−0.04	0.02	−0.08	−0.02

*N* = 489. PROCESS macro by Hayes was used; Model 4 was used for mediation analyses, and interaction effects were estimated using Model 1. SE = standard error; bootstrap samples = 10,000.

### Results discussion: Study 1

We found that EPDR was associated with higher levels of state suppression, which mediated positive effects on both emotional exhaustion and unethical pro-self behavior. We further found that dispositional optimism weakened the positive association between leader-enforced positivity and state suppression, although it did not eliminate the indirect effects. Overall, the positive indirect effects of leader-enforced positivity on exhaustion and unethical behavior via suppression were strongest when dispositional optimism was lower and weaker when optimism was higher.

We also found that HAS was associated with higher levels of state meaning-making and lower levels of state suppression. State meaning-making mediated the negative effects of HAS on both emotional exhaustion and unethical pro-self behavior. We further found that hypercompetitive orientation weakened the positive association between HAS and meaning-making. Overall, the negative indirect effects of HAS on exhaustion and unethical behavior via meaning-making were strongest when hypercompetitive orientation was lower and weaker when hypercompetitive orientation was higher.

### Results and analysis: Study 2

#### Manipulation checks

To assess the effectiveness of the experimental manipulations, we used the HAS and EPDR scales (the same as in Study 1). Participants in the HAS condition perceived their leader as significantly higher in HAS than did participants in the leader-enforced positivity condition, *t*(274) = 16.56, *p* < 0.001 (MHAS = 5.79, *SD* = 0.79 vs. MEPDR = 3.42, *SD* = 1.48). Conversely, participants in the leader-enforced positivity condition perceived their leader as significantly higher in enforced positivity than did participants in the HAS condition, *t*(274) = 6.94, *p* < 0.001 (MEPDR = 4.83, *SD* = 1.49 vs. MHAS = 3.69, *SD* = 1.25). The manipulations were successful.

#### Scenario realism check

To assess scenario realism, participants completed a three-item realism scale adapted from prior vignette-based research (Chen et al., 2011). A sample item is, “It is realistic that I might experience a similar interaction with my leader at work.” Mean scores across items exceeded the midpoint of the scale, ranging from 5.35 to 5.94, with an overall average of 5.70 (*SD* = 0.83). These values are comparable to those reported in prior experimental studies using workplace scenarios (Tang et al., 2023), indicating that participants perceived the scenarios as realistic.

#### Hypothesis testing

A univariate General Linear Model (GLM) revealed a significant effect of condition on state suppression, *F*(1, 269) = 27.51, *p* < 0.001, *ηp^2^* = 0.09. Participants in the leader-enforced positivity condition reported higher suppression (*M* = 4.92, *SD* = 1.12) than those in the HAS condition (*M* = 4.03, *SD* = 1.44) (see Tables 5, 6).

**Table 5:** Tests of between-subjects effects (dependent variable: state suppression).

Source	Type III sum of squares	df	Mean square	*F*	Sig.	Partial eta squared
Corrected model	71.016^a^	6	11.836	7.253	0.000	0.139
Intercept	56.540	1	56.540	34.645	0.000	0.114
Sex	1.252	1	1.252	0.767	0.382	0.003
Age	0.390	1	0.390	0.239	0.625	0.001
Tenure	0.573	1	0.573	0.351	0.554	0.001
Education	2.504	1	2.504	1.534	0.217	0.006
Position	10.609	1	10.609	6.500	0.011	0.024
EPDR × HAS	44.887	1	44.887	27.505	0.000	0.093
Error	439.002	269	1.632			
Total	6027.250	276				
Corrected total	510.018	275				

a *R*^2^ = 0.139 (adjusted *R*^2^ = 0.120).

**Table 6:** Tests of between-subjects effects (dependent variable: state meaning-making).

Source	Type III sum of squares	df	Mean square	*F*	Sig.	Partial eta squared
Corrected model	31.560^a^	6	5.260	2.299	0.035	0.049
Intercept	86.421	1	86.421	37.765	0.000	0.123
Sex	1.408	1	1.408	0.615	0.433	0.002
Age	2.657	1	2.657	1.161	0.282	0.004
Tenure	0.006	1	0.006	0.002	0.961	0.000
Education	0.130	1	0.130	0.057	0.812	0.000
Position	0.096	1	0.096	0.042	0.838	0.000
EPDR × HAS	28.470	1	28.470	12.441	0.000	0.044
Error	615.567	269	2.288			
Total	5749.938	276				
Corrected total	647.127	275				

a *R*^2^ = 0.049 (adjusted *R*^2^ = 0.028).

The results also revealed a significant effect of condition on state meaning-making, *F*(1, 269) = 12.44, *p* < 0.001, *ηp^2^* = 0.04. Participants in the HAS condition reported higher meaning-making (*M* = 4.61, *SD* = 1.56) than those in the leader-enforced positivity condition (*M* = 3.99, *SD* = 1.45).

#### Correlation analysis

Leader-enforced positivity was positively related to state suppression (*r* = 0.30, *p* < 0.01), emotional exhaustion (*r* = 0.18, *p* < 0.01), and unethical pro-self behavior (*r* = 0.25, *p* < 0.01). In contrast, HAS was positively related to state meaning-making (*r* = 0.29, *p* < 0.01) and negatively related to state suppression (*r* = −0.31, *p* < 0.01), emotional exhaustion (*r* = −0.11, *p* < 0.05), and unethical pro-self behavior (*r* = −0.29, *p* < 0.01). State suppression was positively associated with emotional exhaustion (*r* = 0.37, *p* < 0.01) and unethical pro-self behavior (*r* = 0.47, *p* < 0.01), whereas state meaning-making was negatively associated with these outcomes (*r* = 0.23–0.27, *p* < 0.01). As expected, dispositional optimism was positively related to HAS (*r* = 0.24, *p* < 0.01) and state meaning-making (*r* = 0.17, *p* < 0.01) and negatively related to state suppression (*r* = −0.20, *p* < 0.01) and leader-enforced positivity (*r* = −0.13, *p* < 0.01). Hypercompetitive orientation showed the opposite pattern, correlating negatively with HAS (*r* = −0.32, *p* < 0.01) and state meaning-making (*r* = −0.29, *p* < 0.01) and positively with state suppression (*r* = 0.26, *p* < 0.01) (see Table 7).

**Table 7:** Means, standard deviations, and correlations.

Variables	Mean	SD	1	2	3	4	5	6	7	8
Leader-enforced positivity	4.60	1.68	1							
Humility–aspiration signaling	4.26	1.48	−0.08	1						
State suppression	4.47	1.36	0.30^**^	−0.31^**^	1					
State meaning-making	4.29	1.53	−0.02	0.29^**^	−0.27^**^	1				
Dispositional optimism	4.78	1.30	−0.13^**^	0.24^**^	−0.20^**^	0.17^*^	1			
Hypercompetitive orientation	3.50	1.57	−0.05	−0.32^**^	0.26^**^	−0.29^**^	−0.18^**^	1		
Unethical pro-self behavior	4.52	1.36	0.25^**^	−0.29^**^	0.47^**^	−0.24^**^	−0.23^**^	0.07	1	
Emotional exhaustion	4.28	1.39	0.18^**^	−0.11^*^	0.37^**^	−0.26^**^	−0.12	0.19^**^	0.20^**^	1
Sex	1.44	0.49	−0.02	0.06	0.01	0.02	0.04	0.01	0.06	−0.08
Age	37.21	5.15	−0.09	−0.10	0.03	0.06	0.05	−0.02	0.10	0.06
Tenure	8.43	6.11	0.04	0.00	0.07	−0.00	0.03	−0.04	0.02	0.08
Education	1.88	0.753	0.05	−0.12^*^	0.08	0.00	−0.08	0.01	0.01	0.05

N = 276. **p < 0.01, *p < 0.05 (2-tailed). 1 = Leader-enforced positivity, 2 = Humility–aspiration signaling, 3 = State suppression, 4 = State meaning-making, 5 = Dispositional optimism, 6 = Hypercompetitive orientation, 7 = Unethical pro-self-behavior, 8 = Emotional exhaustion.

#### Regression analysis

The results from PROCESS Model 7 indicated that EPDR was positively related to state suppression (*β* = 0.30, SE = 0.05, *p* < 0.001), supporting Hypothesis 1. The interaction between leader-enforced positivity and dispositional optimism was significant (*β* = −0.11, SE = 0.03, *p* < 0.001), indicating that dispositional optimism weakened the positive relationship between leader-enforced positivity and state suppression, consistent with Hypothesis 5a. Leader-enforced positivity was not directly related to state meaning-making (*β* = −0.02, SE = 0.06, *p* = 0.74) (see Table 8).

**Table 8:** Effects of leader-enforced positivity.

Variables	State meaning-making			State suppression			Unethical pro-self behavior			Emotional exhaustion		
	*β*	SE	*p*	*β*	SE	*p*	*β*	SE	*p*	*β*	SE	*p*
Sex	0.05	0.18	0.41	0.05	0.16	0.39	0.02	0.14	0.65	−0.01	0.16	0.82
Age	−0.04	0.02	0.51	−0.03	0.01	0.62	0.04	0.01	0.48	−0.08	0.01	0.154
Education	0.01	0.12	0.93	0.07	0.10	0.22	−0.03	0.09	0.54	0.02	0.11	0.76
Tenure	−0.00	0.01	0.81	0.04	0.01	0.43	−0.01	0.01	0.78	0.05	0.01	0.39
Leader-enforced positivity	−0.02	0.06	0.74	0.30	0.05	0.00	0.10	0.05	0.06	0.01	0.05	0.86
State suppression							0.47	0.05	0.00	0.34	0.06	0.00
Leader-enforced positivity × dispositional optimism				−0.11	0.03	0.00						

Path	Effect	BootSE	BootLLCI	BootULCI
Leader-enforced positivity → state suppression → unethical pro-self behavior	0.14	0.03	0.08	0.21
Leader-enforced positivity → state suppression → emotional exhaustion	0.10	0.03	0.04	0.17

State suppression was positively related to both unethical pro-self behavior (*β* = 0.47, SE = 0.05, *p* < 0.001) and emotional exhaustion (*β* = 0.34, SE = 0.06, *p* < 0.001), supporting Hypotheses 3b and 3a, respectively. Bootstrapped indirect effect analyses revealed that leader-enforced positivity had positive indirect effects on unethical pro-self behavior [indirect effect = 0.14, BootSE = 0.03, 95% CI (0.08, 0.21)] and emotional exhaustion [indirect effect = 0.10, BootSE = 0.03, 95% CI (0.04, 0.17)] via state suppression, supporting Hypothesis 5b.

**Figure 2:**
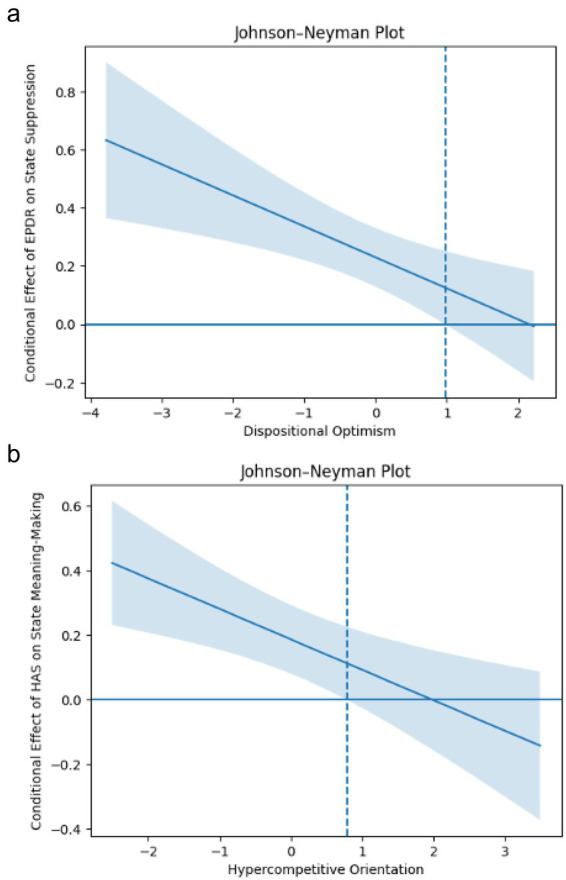
**(a)** Johnson–Neyman plot (EPDR). **(b)** Johnson–Neyman plot (HAS).

The Johnson–Neyman technique was used to examine the moderation effect. The positive effect of leader-enforced positivity on state suppression was significant when dispositional optimism was below 0.98, but became non-significant at higher levels of optimism, indicating that optimism buffers the suppressive impact of enforced positivity (see Figure 2).

#### HAS effects

Results from PROCESS Model 7 indicated that HAS was positively related to state meaning-making (*β* = 0.24, SE = 0.05, *p* < 0.001), supporting Hypothesis 2, and negatively related to state suppression (*β* = −0.29, SE = 0.05, *p* < 0.001). The interaction between HAS and hypercompetitive orientation was significant (*β* = −0.09, SE = 0.03, *p* < 0.001), indicating that hypercompetitive orientation weakened the positive relationship between HAS and state meaning-making, consistent with Hypothesis 6a. HAS was not directly related to emotional exhaustion (*β* = −0.02, SE = 0.05, *p* = 0.79) (see Table 9).

**Table 9:** Effects of humility–aspiration signaling.

Variables	State suppression			State meaning-making			Unethical pro-self behavior			Emotional exhaustion		
	*β*	SE	*p*	*β*	SE	*p*	*β*	SE	*p*	*β*	SE	*p*
Sex	0.03	0.16	0.65	0.07	0.18	0.22	0.04	0.16	0.53	0.02	0.16	0.78
Age	0.01	0.01	0.81	−0.05	0.02	0.35	0.05	0.01	0.37	−0.02	0.02	0.12
Education	0.05	0.11	0.39	0.03	0.12	0.56	−0.01	0.10	0.79	0.05	0.11	0.43
Tenure	0.07	0.01	0.26	−0.02	0.01	0.77	0.02	0.01	0.71	0.07	0.01	0.24
Humility–aspiration signaling	−0.29	0.05	0.00	0.24	0.05	0.00	−0.25	0.05	0.00	−0.02	0.05	0.79
State meaning-making							−0.18	0.05	0.00	−0.26	0.05	0.00
Humility–aspiration signaling × hypercompetitive orientation				−0.09	0.03	0.00						

Path	Effect	BootSE	BootLLCI	BootULCI
Humility–aspiration signaling → state meaning-making → unethical pro-self behavior	−0.04	0.02	−0.08	−0.01
Humility–aspiration signaling → state meaning-making → emotional exhaustion	−0.06	0.02	−0.11	−0.03

State meaning-making was negatively related to unethical pro-self behavior (*β* = −0.18, SE = 0.05, *p* < 0.001) and emotional exhaustion (*β* = −0.26, SE = 0.05, *p* < 0.001), supporting Hypotheses 4b and 4a, respectively. Bootstrapped indirect effect analyses indicated that HAS had negative indirect effects on unethical pro-self behavior [indirect effect = −0.04, BootSE = 0.02, 95% CI (−0.08, −0.01)] and emotional exhaustion [indirect effect = −0.06, BootSE = 0.02, 95% CI (−0.11, −0.03)] via state meaning-making, supporting Hypothesis 6b.

As shown in Figure 2b, HAS was positively related to state meaning-making when hypercompetitive orientation was below 0.79, but this effect became non-significant at higher levels of hypercompetitive orientation, indicating that competitiveness blunts the meaning-enhancing effects of HAS.

## Discussion

This study aimed to explain how EPDR and HAS shape emotional exhaustion and unethical pro-self behavior through distinct psychological pathways. Across a multi-wave field survey and a vignette experiment, we found consistent evidence for the dual-pathway model. In Study 1, EPDR related positively to state suppression, which predicted higher emotional exhaustion and higher unethical pro-self behavior, while HAS was positively related to state meaning-making and negatively to state suppression, which then predicted lower emotional exhaustion and lower unethical pro-self behavior. Dispositional optimism weakened the EPDR → state suppression link, and hypercompetitive orientation weakened the HAS → state meaning-making link. Study 2 complemented these findings by showing that brief episodes of EPDR versus HAS were sufficient to shift state suppression, state meaning-making, emotional exhaustion, and unethical pro-self intentions in the expected directions.

Our findings extend research on emotional labor and display rules. Prior work shows that strict display rules and surface acting drain self-regulatory resources and increase burnout symptoms (Qiu et al., 2024; Chang, 2020). We demonstrate that leader-enforced positive display rules increase state suppression and that this state suppression is a key mechanism linking EPDR to emotional exhaustion and unethical pro-self behavior. This is consistent with evidence that suppression-based emotion regulation is effortful and associated with greater emotional exhaustion (Humphrey et al., 2015). We also show that dispositional optimism softens, but does not remove, the tendency for EPDR to foster state suppression, supporting the idea that optimistic employees interpret controlling signals as less threatening and are somewhat protected from their emotional costs (Diefendorff et al., 2011).

The results also clarify the constructive potential of HAS. Leadership research has linked leader humility to learning and openness (Zhong et al., 2020) and aspirational communication to meaningful work and purpose (Carton et al., 2014). We show that when humility and aspiration are combined as HAS, employees report higher state meaning-making, which is related to reduced emotional exhaustion and reduced unethical pro-self behavior. These results align with evidence that meaningful work lowers burnout and discourages counterproductive or unethical behavior by anchoring conduct in values and long-term goals (Michaelson et al., 2014). However, HAS is not equally effective for all employees. Hypercompetitive orientation weakens the positive effect of HAS on state meaning-making, suggesting that employees who approach work primarily as a zero-sum contest are less likely to translate humility and aspirational goals into a sense of shared purpose (Song, 2021). EPDR and HAS function as distinct social information cues that shape state suppression and state meaning-making in opposite ways, consistent with social information processing theory (Wang et al., 2022).

### Theoretical contributions

Our study contributes to the literature on positive leadership and emotional labor by showing that leader-enforced positivity functions differently from supportive positivity because it operates as a rule about emotional display rather than as interpersonal support. Research on leader humility and affective leadership emphasizes how leader behavior can shape how people coordinate, exchange information, and adapt in teams (Owens and Hekman, 2016; Owens et al., 2013). At the same time, emotional labor research shows that when emotion demands push employees toward suppression-style regulation (surface acting), employees experience greater stress and burnout. Bringing these streams together, our findings suggest that enforced positivity is not simply “more positivity,” but a distinct form of affective control that shifts employees into suppression-based regulation, which then carries downstream costs for wellbeing and ethics.

A second contribution comes from examining HAS as an episodic interactional cue, rather than treating humility primarily as an enduring leader characteristic that accumulates relational benefits over time. Prior work conceptualizes expressed humility as a pattern of observable behaviors that includes receptiveness to feedback (teachability), accurate self-appraisal, and appreciation of others’ strengths (Owens et al., 2013). In team-process accounts, leader humility is similarly framed as a modeled set of behaviors that is socially contagious and organizes teams around owning limitations, appreciating strengths, and pursuing continual improvement and advancement (Owens and Hekman, 2016). Consistent with this logic, our study showed that when leaders combine acknowledging limits with ambitious direction, employees do not merely infer interpersonal warmth; they construct meaning in the moment, which reduces strain and constrains unethical pro-self tendencies.

Our study also contributes to social information processing perspectives by clarifying that leader signals do not operate on a neutral interpretive field. Expressed humility is defined and measured as a social cue that others can observe, including admitting not knowing, acknowledging others’ greater knowledge, recognizing others’ strengths, and being open to advice (Owens et al., 2013). When leaders pair these humility cues with aspirational directional rhetoric, employees must interpret whether the signal grants permission to think and learn or instead constrains expression and channels attention toward display. In this manner, our findings highlight that heterogeneity in employee reactions is not noise; it is the predictable result of interpretive filters that shape whether the same leader cue is construed as meaningful guidance or as affective control.

Finally, our study contributes to the self-regulation and wellbeing literature by clarifying how internal regulation modes connect leader signals to strain and misconduct. In emotional labor research, surface acting is explicitly described as the regulation and suppression of felt emotion and is associated with stress and burnout (Kim, 2020). Building on this logic, our study extends leadership signaling research by showing that leaders can channel employees toward suppression or toward meaning-making, with distinct consequences. When regulation is dominated by suppression, employees bear higher internal costs, and we show that the same “positive” surface behavior can create either meaning or depletion depending on how it is framed and interpreted.

### Practical implications

When teams face uncertain situations or some setbacks, leaders need to know that enforced positivity does not usually work. HAS can be the right choice, because employees interpret enforced positivity as a demand for emotional control rather than support. When leaders acknowledge limits, invite critique, and voice a concrete next step, employees are more likely to construct meaning instead of suppressing emotion. This guidance applies specifically when performance pressure (or error visibility) is high, as these conditions heighten sensitivity to interpretive cues.

In training leaders, a constructive target can be to train them to communicate aspirations without excessive control or *policing.* This allows them to acknowledge constraints and uncertainty, invite corrective input, provide credit, and model teachability. These behaviors map onto leaders expressing humility as a multidimensional skill set. Organizations can assess these behaviors and develop practices to support a more ethical and supportive climate.

It can help employees to treat enforced positivity as a *context signal* about what kinds of expressions are allowed rather than as evidence that their concerns are illegitimate. When the climate cues impression management, people often default to self-censorship, which limits open expression and voice. In contrast, leader-expressed humility signals that such cues can widen expressive latitude rather than constrain it.

### Limitations and future directions

Despite the strengths of the experimental and time-lagged designs, this study is not without limitations. First, the theory specifies an interpretive mechanism, but only optimism and hypercompetitive orientation were modeled as filters. This matters because social information processing depends on how cues are cognitively framed rather than on the cues alone. Future work can extend this mechanism by examining additional interpretive lenses, such as moral attentiveness, voice orientation, regulatory focus, and psychological safety sensitivity.

This study focuses on signals and cues, which limit inference about how suppression and meaning-making evolve across repeated leader interactions. Although we deliberately separated leader signals and mediators across waves to reduce common method bias and capture short-horizon reactions that persisted beyond the immediate episode, this design does not capture longer-term dynamics. Future work can extend the same mechanism using experience-sampling designs to track within-person fluctuations across episodes.

Moreover, to provide additional assurance, future work could consider additional individual-level outcomes that may influence inferences about how suppression and meaning-making aggregate into collective patterns. The theory implies that leader cues communicate social rules, which may become shared constraints. Upon reflection, we suggest that suppression and meaning-making likely scale beyond individual experience. Future work could address this limitation by including our dependent variables alongside other individual factors.

## Conclusion

This empirical study examined the relationships between EPDR, HAS, state suppression, state meaning-making, emotional exhaustion, and unethical pro-self behavior, together with the moderating roles of dispositional optimism and hypercompetitive orientation. Across a multi-wave field survey and a vignette experiment, this study found that EPDR was associated with higher state suppression, which related to greater emotional exhaustion and higher unethical pro-self behavior, whereas HAS was associated with higher state meaning-making and lower state suppression, which related to lower emotional exhaustion and reduced unethical pro-self behavior. This study deepens our understanding of how specific leader signals, rather than broad leadership styles, shape both employee wellbeing and unethical pro-self behavior through distinct psychological pathways.

## Data Availability

The raw data supporting the conclusions of this article will be made available by the authors, without undue reservation.

## Footnotes

1 The prefix state distinguishes both suppression and meaning-making as situationally elicited regulatory responses to the perceived leader signal, rather than stable dispositional tendencies (Lim et al., 2024).
