# Supplementary material for: How leader-enforced positivity and humility–aspiration signaling shape emotional exhaustion and unethical behavior: evidence for distinct psychological pathways
Source: Front Psychol. 2026 May 13;17:1774884. doi: 10.3389/fpsyg.2026.1774884 (PMC13212055; doi:10.3389/fpsyg.2026.1774884)
Supplement: Supplementary file 1 [file Supplementary_file_1.docx]

**S1**

Please imagine you are an employee in a project team. Your supervisor has called a team meeting at a critical stage of the project. Read the following scenario carefully and answer the questions that follow based on the supervisor’s behavior in this meeting.

**Humility–Aspiration Signaling (HAS) Condition**

“The supervisor opens by acknowledging a recent mistake and a personal limitation and asks for critical feedback on the plan. They invite dissent and call on teammates with relevant expertise. After a brief discussion, the supervisor summarizes what was learned and sets a higher, specific performance target for the next cycle. They define a concrete next step, assign clear ownership and a deadline, and explain how the step links to the team’s purpose. The supervisor states confidence that the target is attainable with the team’s input, asks team members to flag risks early, and remains available for questions to hear further advice”.

**Leader-Enforced Positive Display Rules (EPDR) Condition**

“The supervisor opens by stating that the team should maintain a positive tone and that discussing problems is not helpful at this point. When a team member raises a risk, the supervisor redirects attention to “what is going well” and emphasizes avoiding negative comments. The supervisor adds that if anyone feels frustrated or worried, they should keep those feelings to themselves and focus on positive updates. When another team member mentions a delay, the supervisor downplays the issue as minor, minimizes its impact, and reiterates the request to “stay positive.” No request for critical feedback is made, no mistake or limitation is acknowledged, and the meeting ends with a general call to “move forward” without specifying a performance target, next step, owner, or deadline”.

## S2 — Scale Items

### Humility–Aspiration Signaling (HAS)

(Adapted from (65) and (15))

Instructions: The following statements refer to your immediate supervisor's recent behavior. Please indicate your agreement with each statement. (1 = Strongly Disagree, 7 = Strongly Agree)

| **No.** | **Item** |
| --- | --- |
| 1 | Recently, my supervisor actively sought feedback, even if it was critical. |
| 2 | Recently, my supervisor admitted not knowing how to do something. |
| 3 | Recently, my supervisor acknowledged others' greater expertise when relevant. |
| 4 | Recently, my supervisor was open to advice from others. |
| 5 | Recently, my supervisor showed that they expected a lot from us. |
| 6 | Recently, my supervisor would not settle for second best. |
| 7 | Recently, my supervisor set goals for our performance that were quite challenging. |
| 8 | Recently, my supervisor consistently set challenging goals for us to attain. |

### Leader-Enforced Positive Display Rules (EPDR)

(Adapted from (64))

| **No.** | | **Item** | |
| --- | --- | --- | --- |
| 1 | | Recently, my supervisor pushed a positive tone and discouraged frank talk about problems. | |
| 2 | | Recently, my supervisor expected me to suppress negative reactions. | |
| 3 | | Recently, my supervisor expected me to pretend I was not upset or distressed. | |
| 4 | Recently, my supervisor told us to "stay positive" while downplaying real issues. | |  |
